# Atypical HIV-1 Viremia Persistently Detected Exclusively Through the Pol Target by a Dual-Target (Pol and LTR) Assay: A Case Report About a New Diagnostic Challenge

**DOI:** 10.3390/ijms27062595

**Published:** 2026-03-12

**Authors:** Alessandra Amendola, Sara Belladonna, Flavia Smoquina, Giulia Capecchi, Valentina Mazzotta, Maria Grazia Bocci, Fabrizio Maggi, Federica Forbici, Lavinia Fabeni

**Affiliations:** 1 Laboratory of Virology and Laboratories of Biosecurity, National Institute for Infectious Diseases “Lazzaro Spallanzani”—IRCCS, 00149 Rome, Italy; alessandra.amendola@inmi.it (A.A.); sara.belladonna@inmi.it (S.B.); flavia.smoquina@inmi.it (F.S.); fabrizio.maggi@inmi.it (F.M.); lavinia.fabeni@inmi.it (L.F.); 2 Intensive Care Unit, National Institute for Infectious Diseases “Lazzaro Spallanzani”—IRCCS, 00149 Rome, Italy; giulia.capecchi@inmi.it (G.C.); mariagrazia.bocci@inmi.it (M.G.B.); 3 Clinical Department, National Institute for Infectious Diseases “Lazzaro Spallanzani”—IRCCS, 00149 Rome, Italy; valentina.mazzotta@inmi.it

**Keywords:** HIV-1 RNA, HIV-1 viral load, dual-target assay

## Abstract

We report the case of a 48-year-old man admitted with severe pneumonia, profound immunosuppression and multiple co-infections, showing unusual pattern of HIV-1 viremia. With the Aptima HIV-1 Quant Dx assay, a dual-target diagnostic assay for monitoring of viral RNA in people living with HIV-1 (PWH), the patient showed viral RNA consistently detected exclusively through the pol target, while the LTR signal remained absent in all samples during one year of follow-up on antiretroviral therapy. Despite this persistent atypical viral load (pol+/LTR-), near full-length next-generation sequencing of HIV-1 RNA confirmed an almost intact viral genome, including the LTR region and no resistance-associated mutations. Several mechanisms may account for explaining the persistent lack of LTR detection, such as defective quasi-species, RNA structural rearrangements, or epi-transcriptomic modifications interfering with primers annealing, and further studies are currently underway to clarify the biological origins and the clinical implications of the detection patterns of atypical HIV-1 RNA. The case described here is an example of a few PWH undergoing antiretroviral therapy who demonstrated single-target HIV-1 viremia with the Aptima dual-target assay. These particular clinical situations with single-target viremia, even at high levels of HIV-1 RNA, should be carefully considered in clinical management as they would indicate the presence of atypical viral RNA, for which, in some cases, switching antiretroviral therapy could be not necessary.

## 1. Introduction

Monitoring HIV load (i.e., plasma HIV-RNA) in people living with HIV-1 (PWH) represents a fundamental parameter in the clinical management of infection, both to verify the effectiveness of antiretroviral therapy and to monitor the evolution of the disease [1,2]. To quantify HIV-1 RNA there are numerous commercial molecular assays [3,4], mostly based on dual-target detection strategy. In practice, specific primers are employed to detect two distinct regions of the HIV genome. This approach facilitates the concurrent evaluation of both regions, thereby mitigating potential issues caused by mutations that could otherwise hinder accurate identification. The main regions used as targets are the most conserved regions of the viral genome such as pol and LTR, or gag and LTR. To calculate the HIV-1 RNA concentration (i.e., the viral load, VL), most assays use a single fluorescent probe for both the amplified regions of the viral genome, and the VL is extrapolated from the signal intensity through a calibration curve.

Unlike most molecular tests, the Aptima HIV-1 Quant Dx assay (Aptima, Hologic, Inc., San Diego, CA, USA) combines the dual-target detection of pol and LTR with dual-probe detection using two different fluorescent probes: one for each amplified region. In this way, the Aptima yields two VL values on the same plasma sample, one referring to the pol region and the other referring to the LTR region [5]. However, the final VL value reported by the Aptima is usually the one based on the pol amplification, being the most conserved region of the viral genome, considering that the VL value referred to the LTR is usually not significantly different [6].

In this study, we describe the case of a 48-year-old man who showed plasma HIV-RNA exclusively detected through amplification of the pol region, while the LTR target was steadily not detected with the Aptima. Repeated VL determinations with Aptima over one year confirmed HIV-RNA levels > 50 copies/mL persistently detected through the pol target and LTR signal absent.

## 2. Case Presentation

A 48-year-old man was transferred on 14 October 2024, from Isola Tiberina Hospital to the Intensive Care Unit of the National Institute for Infectious Diseases (INMI) Lazzaro Spallanzani, due to severely compromised clinical conditions, characterized by interstitial pneumonia.

**Figure 1:**
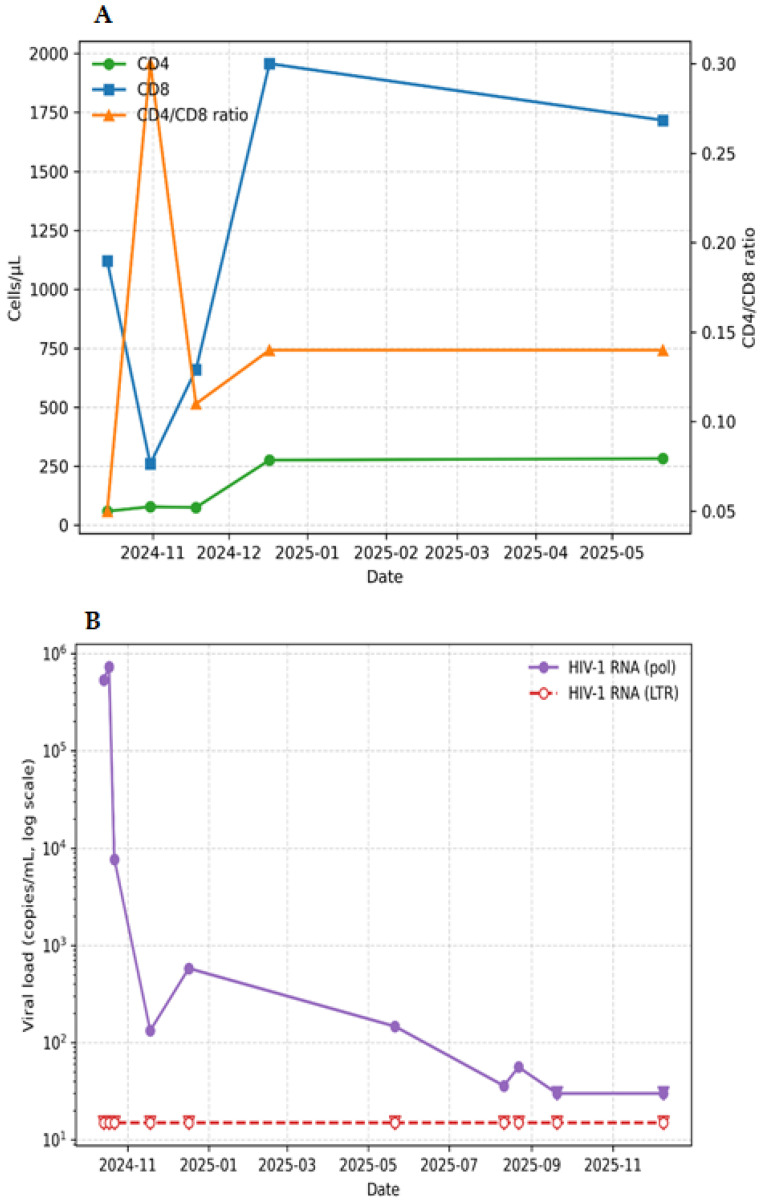
(**A**,**B**): Immunological parameters and HIV-1 RNA values observed during follow-up. (**A**) Immune reconstitution markers. The green line represents the trend in the number of CD4+ T lymphocytes; the blue line represents the number of CD8+ T lymphocytes; the orange line represents the CD4/CD8 ratio. (**B**) HIV-1 RNA (copies/mL). The purple line shows the trend of viral load detected with the pol target by the Aptima system. At each measurement, the viral load related to the LTR target was always undetected (as shown by the red line).

At admission, the patient showed profound immunosuppression, with 59 cells/mm^3^ CD4 T cells and a 0.050 CD4/CD8 ratio (Figure 1A). Human immunodeficiency virus 1 (HIV-1) infection, already known at admission, was confirmed through serological screening and immunoblot testing. HIV-1 VL, measured with the Aptima, was 534,708 copies/mL HIV-1 RNA (Figure 1B). At the same time, various microbiological and virological tests were performed (Table 1), revealing multiple co-infections. Cytomegalovirus (CMV) was detected both in whole blood (167,812 copies/mL) and bronchoalveolar lavage (BAL) fluid (1031 copies/mL). Human Herpes 8 (HHV-8) viremia was 7967 copies/mL, and the SARS-CoV-2 antigen test resulted positive (COI: 20.66). Total antibodies for *Treponema pallidum* were positive. BAL fluid culture test for common germs further showed the presence of *Aspergillus* spp. and *Pneumocystis jirovecii*.

### 2.1. Follow-Up

During hospitalization, the patient experienced additional infectious complications (Table 1).

Four days after admission, *Staphylococcus aureus* was isolated from BAL fluid. On 22 October 2024, both multidrug-resistant (MDR) and KPC-producing *Klebsiella pneumoniae* were identified initially confined to BAL and then also detected in blood hemocultures. In the following month, on 18 November, *Candida albicans* was isolated from BAL in addition to the already confirmed presence of *K. pneumoniae*.

From a virological perspective, four days after admission, repetition of tests confirmed active replication of CMV and HHV-8, showing viral loads of 88,059 copies/mL and 9414 copies/mL, respectively. Epstein–Barr virus (EBV) was also detected with viremia of 299,372 copies/mL. Given the degree of deep immunosuppression, Torque Teno virus (TTV) was also assessed in the blood as an additional surrogate marker of immune dysfunction [7], showing a markedly elevated value of 9,312,300 copies/mL, while plasma HIV-1 RNA in the same sample was 729,472 copies/mL (Figure 1B).

On 22 October, a subsequent evaluation of viral pathogens showed progressive decline in CMV (25,201 copies/mL) and EBV (185,535 copies/mL) loads, whereas TTV viremia further increased to 11,066,130 copies/mL, thus confirming persistent immunosuppression. By 6 November, CMV viremia decreased to 500 copies/mL, with a transient rebound on 11 February 2025 (732 copies/mL), and became persistently undetectable from 20 September 2025. HHV-8 was reassessed on 17 December, showing persistent low-level viremia (4212 copies/mL).

During hospitalization, the patient received the following medications favoring the gradual restoration of immune function and general state of health. The antiretroviral therapy (ART) with Biktarvy (Bic/FTC/TAF) was initiated, while antibacterial treatment included ceftriaxone, meropenem/vaborbactam, daptomycin and trimethoprim–sulfamethoxazole, according to microbiological findings and clinical evolution. Adjunctive corticosteroid therapy with methylprednisolone was administered, together with antifungal therapy based on caspofungin and antiviral therapy with ganciclovir.

### 2.2. HIV-1 Viremia and Next-Generation Sequencing (NGS) of Plasma HIV-1 RNA

HIV-1 viremia was monitored with the Aptima HIV-1 Quant Dx diagnostic assay, based on dual-target (pol and LTR) and dual-probe technology. The method integrates target capture, isothermal TMA, and real-time fluorescent detection to obtain HIV-1 RNA quantification. The viral RNA in plasma samples is released by chemical lysis and hybridizes to capture specific oligonucleotides immobilized on magnetic particles. Amplification is then achieved via TMA using reverse transcriptase and T7 RNA polymerase. Fluorescent probes hybridizing to amplicons enable real-time detection of amplification products and the time taken for the fluorescent signal to reach a specified threshold (tTime) is proportional to the initial concentration of the HIV-1 RNA targets in the sample [5]. Although the tTime of both targets is recorded, the Panther software (version 7.4.3.0) returns the result of viremia related to the target pol. If the pol region is not detected but LTR target is quantified, the HIV-1 RNA value released by the software refers to the LTR-detected viremia; if both the target regions result not-detected, the result of viremia is HIV-1 RNA not-detected [6].

Throughout the observation period, patient’s HIV-1 viremia remained detectable only through the pol target, while the LTR was repeatedly undetected (Figure 1B). During the following 14 months, HIV-1 viremia decreased from 729,472 to <30 copies/mL with a CD4 count increase to 276 cells/mL, although CD4/CD8 ratio was rather low, between 0.110 and 0.300 (Figure 1A). On 18 November 2024, plasma HIV-1 RNA fell to 133 copies/mL, and from the discharge from the hospital on 7 December 2024, it oscillated between 580 and 55 copies/mL, slowly reaching <30 copies/mL in December 2025, always detected exclusively with the pol region (Figure 1B).

The first sample collected for measurement of HIV viremia was used to perform next-generation near full-length (NFL) sequencing of viral RNA (approximately from nucleotides 1 to 9719 of the HXB2 genome [8]) carried out on the Ion GeneStudio S5 prime System platform (Thermo Fisher, Waltham, MA, USA) by using the Ion AmpliSeq WGS HIV-1 custom panel (Thermo Fisher, Waltham, MA, USA). NFL-NGS analysis of the viral genome showed an intact viral genome in the plasma sample, including the LTR region not detected by Aptima. Subtype B was determined by phylogenetic analysis on the consensus sequence obtained by NFL sequencing [9]. No resistance-conferring mutations or deletions were observed in the pol region (nucleotides 2253–5096 of the HXB2 genome) and in the LTR region. In particular, the average read coverage was 2221, with a nucleotide conservation of 78.9% of the consensus sequence relative to the reference sequence, and 6292, with a conservation of 76.7% for LTR and pol, respectively.

## 3. Discussion

We report the case of an HIV-1 infected ART-treated man who persistently showed atypical HIV-1 RNA VL detectable exclusively through the pol target with the Aptima dual-target assay, while LTR was repeatedly not detected. Despite the persistent lack of LTR amplification during follow-up, NFL-NGS of HIV-1 RNA confirmed the presence of an almost-intact LTR region.

Various hypotheses can be formulated to explain this contradictory result. One could be the presence of mutations within the LTR primer-binding site targeted by the Panther system which could prevent detection and amplification of this region, although the Hologic company states the use of multiple primers covering the entire LTR region to reduce this eventuality [5].

Another explanation may be viral quasi-species coexistence: the patient could harbor a mixed population of HIV-1 variants, including both intact genomes and LTR-defective forms. In this scenario, the Aptima assay might detect only defective variants lacking the LTR probably more numerous, whereas deep sequencing would still identify minority variants with an intact LTR, having its higher sensitivity.

Alternatively, epi-transcriptomic modifications of viral RNA (such as methylation) may impair primer hybridization, thus preventing the LTR detection. In this case, further analysis would be necessary to verify the presence of posttranscriptional modifications of the HIV-1 RNA at the LTR region in these patients, such as RNA methylation modifications, including m6A (N6-methyladenosine), m5C (5-methylcytidine), m7G (N7-methylguanosine) and 2-O-methylation [10,11]. Numerous studies have shown that the RNA of HIV-1 undergoes modifications during infection influencing the stability, metabolism, translation, splicing, and in some cases promoting viral replication [12]. In addition, recent studies demonstrated that the HIV-1 LTR region can fold into complex secondary structures, including stem-loop formations and dimerization interfaces, which may interfere with primer accessibility and amplification [13]. In parallel, epi-transcriptomic analyses of the HIV-1 LTR have identified N6-methyladenosine (m6A) modifications, a modification implicated in RNA packaging and cellular incorporation. The presence of such modifications could similarly interrupt primer annealing and lead to amplification failure [14], determining the atypical viremia detected by Aptima.

Beyond the hypotheses advanced above, it is worth considering that detection of HIV-1 RNA exclusively through a single target with a double-target test can be possible with Aptima, as it uses two different fluorescent probes, one for each viral target. In the past, thanks to this analytical configuration of Aptima, we described a small group of PWH on effective ART who showed VL not detected with pol but detected and quantified at high levels with the second target LTR [6,15]. Further analyses of these LTR-detected elements led us to the conclusion that HIV-1 RNA was detected only through the LTR consisted of incomplete, defective viral RNA genomes, likely lacking the intact pol sequence and included in aberrant particles varying in size, shape, and nucleoid morphology, with a significantly lower diameter [15].

From our long experience with PWH showing HIV-1 viremia detected only with the LTR target or those others with viremia detected exclusively through the pol target, we know that in both cases these are people taking effective ART and showing microbiological and immunological clinical parameters typical of people on ART with VL undetected with both targets ([15] and additional unpublished data). Therefore, it is likely that the single-targeted VL detected by Aptima are made of HIV-1 RNA that is either incomplete (i.e., derived from defective integrated viral genomes), or post-transcriptionally modified (i.e., by epigenetic changes), or rearranged in their secondary and/or tertiary structure in non-canonical forms, and therefore unable to anneal with Aptima primers.

Importantly, from a clinical management perspective, single-targeted VL detected with Aptima, even at high concentrations, would represent viral genomes incapable of producing viral progeny through new cycles of infection and viral replication due to alterations in sequence or structural conformation of HIV-1 RNA. Therefore, these PWH on ART, who exhibit persistent atypical HIV-1 viremia with the Aptima dual-target assay, need to be carefully evaluated before considering a possible switch of ART.

## 4. Conclusions

In conclusion, here we described the case of a PWH admitted to the intensive care unit with multiple co-infections (*K. pneumoniae*, *Aspergillus* spp., *P. jirovecii*, *C. albicans*) who persistently showed atypical HIV-1 RNA loads throughout clinical follow-up. HIV-1 RNA was steadily detected with only one target (pol) with dual-target diagnostic system although intact viral genome was revealed by NFL-NGS. All these findings led us to believe that the patient’s clinical history was primarily determined by *K. pneumoniae* and other infections, rather than reactivation of HIV-1 replication. Further studies on the course of the HIV disease in PWH with pol-detected and LTR not-detected atypical viremia are currently underway.

## Figures and Tables

**Table 1:** Microbiological agents detected during hospitalization. Abbreviation’s list: ND: not detected. HIV-1: Human Immunodeficiency virus 1. CMV: Citomegalovirus. EBV: Epstein–Barr virus. HHV-8: Human Herpes virus 8. TTV: Torque Teno virus. SARS-CoV-2: Severe Acute Respiratory Syndrome COronaVirus 2. BAL: Broncho Alveolar Lavage.

Date	CMV (Copies/mL)	EBV (Copies/mL)	HHV-8 (Copies/mL)	TTV (Copies/mL)	SARS-CoV-2 Antigen (COI)	Bacteria	Mycosis
14/10/24	1031 (BAL); 67,812 (Blood)	–	7967	–	20.66	*Treponema pallidum* (serum)	*Aspergillus* spp. (BAL); *P. jirovecii* (BAL)
18/10/24	88,059	299,372	9414	9,312,300	Negative	–	–
22/10/24	25,201	185,535	–	15,066,130	–	*K. pneumoniae* (BAL)	–
28/10/24	–	–	–	–	–	*K. pneumoniae* (BAL)	–
31/10/24	–	–	–	–	–	*S. aureus* (BAL)	–
06/11/24	<500	<500	–	–	–	*K. pneumoniae* (Blood and BAL)	–
11/11/24	–	–	–	–	–	*K. pneumoniae* (BAL)	–
18/11/24	–	–	–	–	–	–	–
17/12/24	513	–	4212	–	–	*K. pneumoniae* (BAL)	*C. albicans* (BAL)
21/05/25	732	–	–	–	–	–	–
11/08/25	–	–	–	–	–	–	–
22/08/25	–	–	–	–	–	–	–
20/09/25	ND	ND	–	–	–	–	–
09/12/25	ND	ND	–	–	–	–	–

## Data Availability

The data presented in this study are available on request from the corresponding author. The data are not publicly available because they are stored in a laboratory management system accessible only to permanent staff.

## Abbreviations

HIV-1	Human Immunodeficiency virus 1
PWH	People with HIV
CMV	Citomegalovirus
EBV	Epstein–Barr virus
HHV8	Human Herpes virus 8
TTV	Torque Teno virus
SARS-CoV-2	Severe Acute Respiratory Syndrome COronaVirus 2.
ART	Antiretroviral therapy
NFL	Near full-length
NGS	Next-generation sequencing
BAL	Broncho Alveolar Lavage
